# 461. Classical Antigen Presenting Cell Activation Correlates with T Cell Immunity and COVID-19 Severity

**DOI:** 10.1093/ofid/ofab466.660

**Published:** 2021-12-04

**Authors:** Zhongyan Lu, Jarina Pena-DaMata, Katherine Pohida, Camille Lake, Nusrat J Epsi, Stephanie A Richard, Brian Agan, Simon Pollett, Mark P Simons, Clifton Dalgard, Paul W Blair, Josh Chenoweth, Andrew L Snow, Timothy Burgess, Allison M Malloy

**Affiliations:** 1 Uniformed Services University of the Health Sciences, Bethesda, Maryland; 2 Uniformed Service University of the Health Sciences, Bethesda, Maryland; 3 HJF, Bethesda, Maryland; 4 Infectious Disease Clinical Research Program, Department of Preventive Medicine and Biostatistics, Uniformed Services University of the Health Sciences, Bethesda, MD and Henry M. Jackson Foundation, Bethesda, MD, Bethesda, Maryland; 5 Infectious Disease Clinical Research Program, USU/HJF, Bethesda, Maryland; 6 The American Genome Center, Uniformed Services University of the Health Sciences, Bethesda, Maryland; 7 Uniformed Services University, Bethesda, Maryland; 8 Henry M. Jackson Foundation, Bethesda, Maryland; 9 Infectious Disease Clinical Research Program, Bethesda, Maryland

## Abstract

**Background:**

The initial response of immune cells against respiratory viruses often determines the severity and duration of disease. The early trajectory of the immune response during infection with SARS-CoV-2 remains poorly understood. Dysregulation of innate immune factors that facilitate viral clearance and the adaptive response, such as type I interferons, have been implicated in severe COVID-19. However, collection of biological samples during the first seven days post-symptom onset has posed a logistical challenge, limiting our knowledge surrounding the immune responses that drive protection versus immunopathology.

**Methods:**

From March 2020, Military Health System beneficiaries presenting with a positive SARS-CoV-2 test, a COVID-19 like illness, or a high-risk SARS-CoV-2 exposure at nine military medical treatment facilities across the United States were eligible for enrollment in our longitudinal cohort study, which included collection of respiratory sample, sera, plasma, and peripheral blood mononuclear cells (PBMCs). Twenty-five SARS-CoV-2 infected study participants provided samples with in the first seven days of symptom onset, fifteen of whom were hospitalized with COVID-19. We employed multiparameter spectral flow cytometry to comprehensively analyze the early trajectory of the innate and adaptive immune responses.

**Results:**

We discovered that early activation of critical antigen presenting cell subsets was impaired upon comparing inpatients with outpatients, correlating with decreased antigen-experienced T cell responses. Specifically, we noted reduced expression of key costimulatory molecules, CD80 and CD86, on conventional dendritic cells that are required for viral antigen-specific T cell priming. Reduction in CD38, a marker of activation was also observed on inpatient dendritic cell subsets.

**Conclusion:**

Reduced antigen presenting cell activation and expression of ligands that facilitate T cell engagement may impede the efficient clearance of SARS-CoV-2, coinciding with more severe disease in our cohort. Further analysis of the functional activation of early innate immune responses triggered by SARS-CoV-2 may unveil new immune biomarkers and therapeutic targets to predict and prevent severe disease associated with inadequate T cell immunity.

**Disclosures:**

**Simon Pollett, MBBS**, **Astra Zeneca** (Other Financial or Material Support, HJF, in support of USU IDCRP, funded under a CRADA to augment the conduct of an unrelated Phase III COVID-19 vaccine trial sponsored by AstraZeneca as part of USG response (unrelated work))

